# Medieval Spanish (12th–15th centuries) named entity recognition and attribute annotation system based on contextual information

**DOI:** 10.1002/asi.24399

**Published:** 2020-08-19

**Authors:** Mª Luisa Díez Platas, Salvador Ros Muñoz, Elena González‐Blanco, Pablo Ruiz Fabo, Elena Álvarez Mellado

**Affiliations:** ^1^ UNED. POSTDATA Project ERC Starting Grant. Laboratorio de Innovación en Humanidades Digitales Universidad Nacional de Educación a Distancia Madrid Spain; ^2^ CoverWallet. POSTDATA Project ERC Starting Grant. Laboratorio de Innovación en Humanidades Digitales Madrid Spain

## Abstract

The recognition of named entities in Spanish medieval texts presents great complexity, involving specific challenges: First, the complex morphosyntactic characteristics in proper‐noun use in medieval texts. Second, the lack of strict orthographic standards. Finally, diachronic and geographical variations in Spanish from the 12th to 15th century. In this period, named entities usually appear as complex text structure. For example, it was frequent to add nicknames and information about the persons role in society and geographic origin. To tackle this complexity, named entity recognition and classification system has been implemented. The system uses contextual cues based on semantics to detect entities and assign a type. Given the occurrence of entities with attached attributes, entity contexts are also parsed to determine entity‐type‐specific dependencies for these attributes. Moreover, it uses a variant generator to handle the diachronic evolution of Spanish medieval terms from a phonetic and morphosyntactic viewpoint. The tool iteratively enriches its proper lexica, dictionaries, and gazetteers. The system was evaluated on a corpus of over 3,000 manually annotated entities of different types and periods, obtaining F1 scores between 0.74 and 0.87. Attribute annotation was evaluated for a person and role name attributes with an overall F1 of 0.75.

## 1 INTRODUCTION

Named Entity Recognition or NER (Nadeau & Sekine, 2007) consists in identifying text spans called Named Entities (NE), which refer to a set of categories relevant for information needs in a given application domain. We may be generically interested in the names of people or organizations mentioned, on locations or in the names of works of art and artistic techniques. In all cases, identifying NEs provides a useful basic overview of the content of the text.

NER is seen as a primary task in the Information Extraction field (Grishman, 2015), the goal in this field being to turn unstructured text (i.e., unannotated text) into structured data reflecting the content of the text. NER has been applied to a wide range of domains, from newswires (Tjong Kim Sang & De Meulder, 2003) to microblogs (Ritter, Clark, Mausam, & Etzioni, 2011; Strauss, Toma, Ritter, de Marneffe, & Xu, 2016) to biomedical literature (Collier, Ruch, & Nazarenko, 2004). Applying NER in humanities texts is becoming more relevant nowadays, as more digitized corpora are becoming available in the humanities (Ehrmann, Colavizza, Rochat, & Kaplan, 2016).

This work focuses on NER in a very specific scenario, namely Medieval Spanish texts covering genres like legal documents, epic poetry, narrative, or drama. This application case shows particular challenges, which are only partially addressed in existing systems for this language's variety (Iglesias Moreno, Aguilar‐Amat, & Sánchez Cuadrado, 2014). As a first challenge, the difficulties generally encountered by natural language processing (NLP) tools and technologies when dealing with historical language varieties (Sporleder, 2010): Medieval Spanish lacked orthographic normalization, which results in variability in the way the same lexical items get written. Accordingly, coverage in lexical resources can only be imperfect. The use of capitalization was also unstable in Medieval Spanish. This feature is a challenge for NER since NEs are largely proper nouns. The third hurdle for Medieval Spanish NER is diachronic evolution in the language throughout the medieval period (Alvar, 1996). Finally, entities in Medieval Spanish often occur as complex structures, complemented with entity attributes or showing other embedded entities within them. Person names often appear together with their nicknames or with formulaic language attached to them, with nobility or professional titles, or information about a person's geographic origin. For instance, consider the following NE, which shows a complex structure—The English gloss shows standardized spelling besides a translation (1):

(1) myo c'id Ruy diaz de biuar

Mine Cid (Lord) Ruy Diaz of Bivar

Instead, it is proposed that an informative way to represent the NE's internal structure be along the following lines (2):

(2) [_*persName*_ [_*addName*_myo c'id] Ruy diaz de [_*placeName*_ biuar]]

[_*persName*_ [_*addName*_ mine Cid] Ruy Diaz of [_*placeName*_ Bivar]]

The person name itself could be argued to be *Ruy diaz de biuar* (or perhaps solely *Ruy diaz*). However, there is a nickname *myo c'id*, based on an Arabic way to say *My Lord*, prepended to that entity. Also, the person's birthplace occurs as part of the person's name. It would be useful if a NER system could annotate this rich structure. Note that example (1) also shows some of the challenges mentioned above, like lack of standards and unstable capitalization: for example, *biuar* can also appear capitalized and sometimes occurs as *bivar*, and the same person is sometimes referred to as *Rodrigo* rather than *Ruy*.

Our system is a symbolic system based on entity detection rules (compiled as automata) and lexical resources for Medieval Spanish, some of which (e.g., a verb subcategorization dictionary) are new resources created for the system. Non‐standard orthography was handled thanks to a variant generator which takes into account historical linguistic patterns of morphophonological evolution to map textual forms to the system's lexical resources.

As regards the demands posed by the internal structure of medieval entities, the system's contributions are two: First, it is geared towards a custom entity taxonomy, intended to represent medieval entities in a way useful for humanities scholars (Álvarez Mellado et al., 2020). Second, entity attributes (or entity structure) are analyzed via dependency parsing.

### 1.1 *Related work*

NER has been a very active area of research (see Agerri & Rigau, 2016; Nadeau & Sekine, 2007 for a review). However, NER for less‐resourced language varieties poses specific challenges. We discuss briefly here annotation schemes and technological approaches used for NER as relevant to our work. We will also address the scarce existing literature on Medieval Spanish NER.

### 1.2 *Entity annotation schemes*

Several annotation schemes or taxonomies for named entities have been developed and applied extensively, partly thanks to evaluation campaigns in the Information Extraction field. Some of the taxonomies developed cover only a few broad entity types, such as the one in the Message Understanding Conference‐6 task, (MUC) (Grishman & Sundheim, 1996) with six types: people, organizations, locations, time, currencies, and percentages. Another seminal task took place at the Conference on Computational Language Learning (CoNLL) in 2003 (Tjong Kim Sang & De Meulder, 2003). The typology covered similar categories, besides a miscellaneous category with several new types. Much more detailed taxonomies have also been developed, like Sekine's extended entity hierarchy, with over 200 types (Sekine & Nobata, 2004). Other researchers adopt a more flexible approach, proposing both a coarse‐grained taxonomy that can be used directly for tagging, as well as subtypes for each of the coarse types (Desmet & Hoste, 2014). Another relevant characteristic of most annotation schemes is that they do not allow nested entities, one exception being guidelines developed within the ACE (Automatic Content Extraction) campaigns (Linguistic Data Consortium, 2008). We consider, however, that nested entities are informative to analyze medieval texts. These texts tend to provide detail about person names, such as titles (nobility or authority or religious titles), nicknames, or family and geographic origin information. Nested entities are useful to annotate such information. This feature, coupled with other features of medieval entities, led us to propose a new entity taxonomy. The entity types we allow for can be seen as conceptually close to structured named entities (Ringland, 2015; Rosset et al., 2012), given that nested entities are permitted in our typology.

### 1.3 *NER technology*

Regarding technological approaches to NER, expectably, general trends in NLP apply to NER too. Early systems in the 1990s, during the MUC campaigns (Chinchor & Marsh, 1998) used finite‐state technology, for example, FASTUS (i.e., Finite State Automaton Text Understanding System) (Hobbs et al., 1993). By the CoNLL 2003 task, supervised learning was the dominant approach; commonly used models have been Conditional Random Fields (Lafferty, McCallum, & Pereira, 2001) and Support Vector Machines (Cortes & Vapnik, 1995). Unsupervised systems have been developed as well (Cucchiarelli & Velardi, 2001) and current NER tools use deep recurrent neural networks (Huang, Xu, & Yu, 2015). For language varieties or domains with few linguistic resources, where the annotation effort would be larger, symbolic systems have nevertheless been employed even recently. (Borin, Kokkinakis, & Olsson, 2007) used a rule‐based system working on historical varieties of Swedish. Using symbolic approaches has also been frequent in humanities applications. (Grover, Givon, Tobin, & Ball, 2008) chose to implement rule‐based systems for performing NER on digitized 17th and 19th century Parliament records, arguing that unusual orthography and lack of applicability of available PoS‐taggers to the material would make supervised learning inefficient. (Volk et al., 2010) built multilingual rule‐based NER systems focusing on person and geographical names in Alpine heritage corpora. The Edinburgh geoparser, which disambiguates geographical names against a gazetteer, uses a rule‐based NER module for identifying geographical names (Alex, Byrne, Grover, & Tobin, 2015; Grover et al., 2010). More recently (Thomas & Sangeetha, 2019) developed a hybrid NER system integrating rule‐based deep‐learning and clustering‐based components, in order to extract generic entity types (person, location, and organization) in domains which lack labeled datasets.

### 1.4 *Medieval Spanish NER*

Previous studies on automatic extraction of named entities in Hispanic Medieval texts (Iglesias Moreno et al., 2014) were carried out using the *Freeling* tool, (Padró & Stanilovsky, 2012),^1^ which includes data sources for Old Spanish. With this tool, entities that appear isolated in the text or that show a simple syntactic structure are properly recognized. However, entities with a complex structure and other specificities of medieval entities, pose problems for the tool. We discuss these issues below based on examples.

**FIGURE 1:**
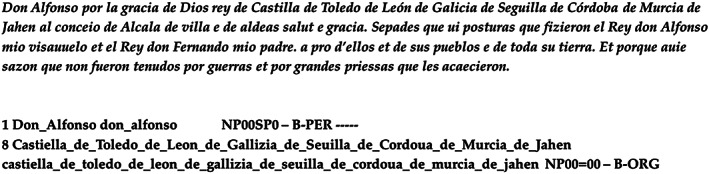
Notarial text of the Alfonsi period and Named Entity Recognition results by the Freeling toolkit

**FIGURE 2:**
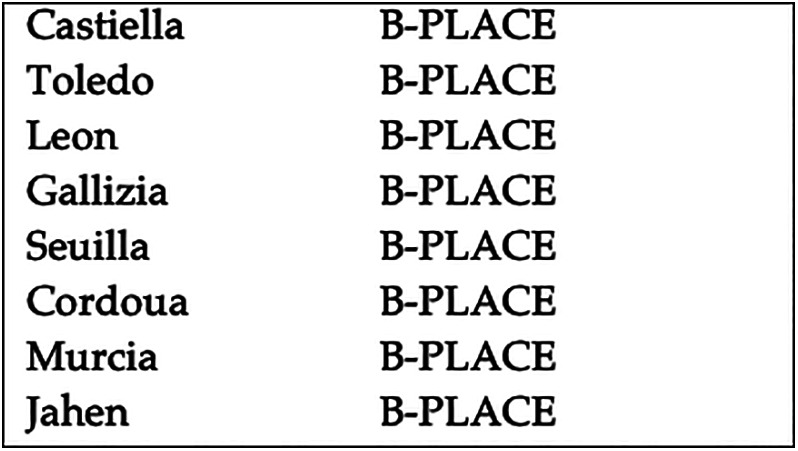
Correct entity segmentation for the first sentence from Figure 1

For instance, Figure 1 shows (top) a sequence of place names, joined with the preposition *de* (*of*), without further orthographic separators between them. Freeling's NER results for the sequence are also displayed (mid and bottom). Given the lack of orthographic delimiters, Freeling considers the complete sequence as a single entity, tagging it as an organization, instead of extracting each place name separately. Figure 2 shows what the correct place name recognition results would be.

Variation in the use of character case for proper nouns also poses difficulties for standard tools like Freeling. This tool tags the sequence *[Dd]on Alfonso* differently whether the form of address *Don* appears with an uppercase or lowercase initial; in the first case, the form of address is segmented as part of the person name, but not in the second case.

## 2 NAMED ENTITIES IN MEDIEVAL SPANISH AND OUR ENTITY TYPOLOGY

This section presents some characteristics of Medieval Spanish and of Named Entities in this language variety followed by the new entity taxonomy we propose to annotate them.

### 2.1 *Medieval Spanish and named entities*

Several characteristics of Medieval Spanish require specific treatment. Regarding the surface form of lexical items, custom lexical resources are required, given the orthographic variation in the absence of a written norm or due to diachronic evolution (Alvar, 1996). In addition, the complex syntactic structures in which entities (particularly person names) get realized also need an appropriate solution.

An example of widespread variation in the surface representation of a given lexical item would be the different variants for the name of the city of Seville, such as *Seville*, *Seuilia*, *Seuilla*, and others. Absence of these variants in the gazetteers and lexica available to treat medieval text requires applying variant generation rules, to find the closest in‐vocabulary item among the available lexical resources. A factor compounding this problem is, as (Cano‐Aguilar, 2004), argued, the fact that phonetic changes do not operate with the same regularity in named entities as in the rest of the lexicon. Besides, the use of capitalization with proper nouns was also irregular, as pointed out by (Albaigès & i Olivart, 1995).

In medieval texts, entities are often accompanied by or occur within enumerations of names with no punctuation marks at all that would help in the delimitation of recognizable entity‐constituents. Nobility titles are often prepended or appended to a person's name, as well as geographical locations related to that person's titles. Role names related to political functions often accompany person names in medieval texts. Nicknames or formulaic language also often co‐occur with entities like person names, saints or deities. The sequence in (3) is an example (with a gloss in English).

(3) Don Alfonso por la gracia de Dios rey de Castiella de Toledo de Leon

Don Alfonso by the grace of God king of Castilla of Toledo of Leon

de Gallizia de Seuilla de Cordoua de Murcia e de Jaen

of Galicia of Seville of Cordoba of Murcia and of Jaen

### 2.2 *Proposed *entity* taxonomy*

Our entity typology is a TEI‐based (i.e., text encoding initiative) annotation scheme for medieval entities trying to respond to the needs of literary scholars, historians, or other humanists (Álvarez et al., 2020).

Based on that typology, the system detects the following entity types (and subtypes where applicable):

- ***persName***: Person names, covering first names, surnames, or family names. For example, *Celestina* or *Rodrigo Díaz*. Several subtypes are defined:
- ***deity***: names of saints and divinities, such as *Dios* (*God*) or *Cupido* (*Cupid*).
- ***nickname***: person nicknames, when they appear in isolation, for example, *El Campeador* (*The Warrior*), which was a nickname for Rodrigo Díaz de Vivar, a military leader in Castile. When nicknames appear complementing the actual person name, the *addName* type is used instead
- ***nickname*_*deity***: nicknames for saints or divinities, such as *Rey de Reyes* (*King of Kings*) for God.
- ***addName***: This type identifies nicknames when they are used in adposition to the “official” name of a person. For example, the underlined sequence in *Pedro el Cruel* (*Peter the Cruel*).
- ***placeName***: Geopolitical units such as countries, cities, towns, regions, so on, such as *Salamanca* or *Castiella* (a medieval variant for *Castilla*, *Castile* in English) A subtype is defined:
  - ***facilities***: Buildings or monuments, like castles, monasteries, or bridges, such as *Castillo de Ella* (*Castle of Ella*).
- ***geogName***: geographic features like rivers, mountains, deserts, oceans, so on. For example, *río Tormes* (*Tormes river*).
- ***orgName***: organization names like religious orders, armies or governmental institutions, for example, *corona de Castilla* (*Crown of Castile*).
- ***roleName***: In case, they complement a person's name, they can be seen as attributes of that person. For instance, the underlined sequence in *Alfonso*, *rey de Castilla* (*Alfonso*, *King of Castile*) expresses the role of Alfonso as an authority (the King). However, role names can also appear on their own; *King of Castile* could also appear by itself. We have defined three subtypes for the *roleName* type:
  - ***honorific***: These do not appear by itself. Rather, they are forms of address like *don*, *seor* (similar to “*Mr*.,” for men), or *donna* (for women).
  - ***family***: Family relationships. We judge these relevant for the study of medieval texts, for historical purposes. The family origin of nobility and rulers is often mentioned in these texts. When family information is given for a person, this is attached to the person within a *family* tag, thanks to dependency parsing. For instance, in *Alfonso*, *filo de Juan y Maria* (*Alfonso*, *son of Juan and Maria*), the underlined sequence would be tagged as a *family* type.
  - ***authority***: It identifies ruler roles such as *rey* (*king*) or *obisspo* (*bishop*), attaching to the role its jurisdiction, diocese or geographical area over which this authority extends.

Note that some of our types could be considered as entity attributes, for example, types indicating family relations like “son of,” and it could be argued that our taxonomy, thus, incorporates an element of relation extraction rather than simply NER. In any case, these attributes are informative about the entities they are part of and we wanted to ensure their extraction.

## 3 ARCHITECTURE OF THE MEDIEVAL SPANISH NER SYSTEM

The goal of the system was identifying named entities as well as some of their attributes, in the latter case via dependency parsing. Annotated data for such a task were unavailable, and it would be costly to produce manual annotations to train a statistical model for the task. Accordingly, we relied on hand‐crafted rule‐sets supplemented by custom lexical resources.

**FIGURE 3:**
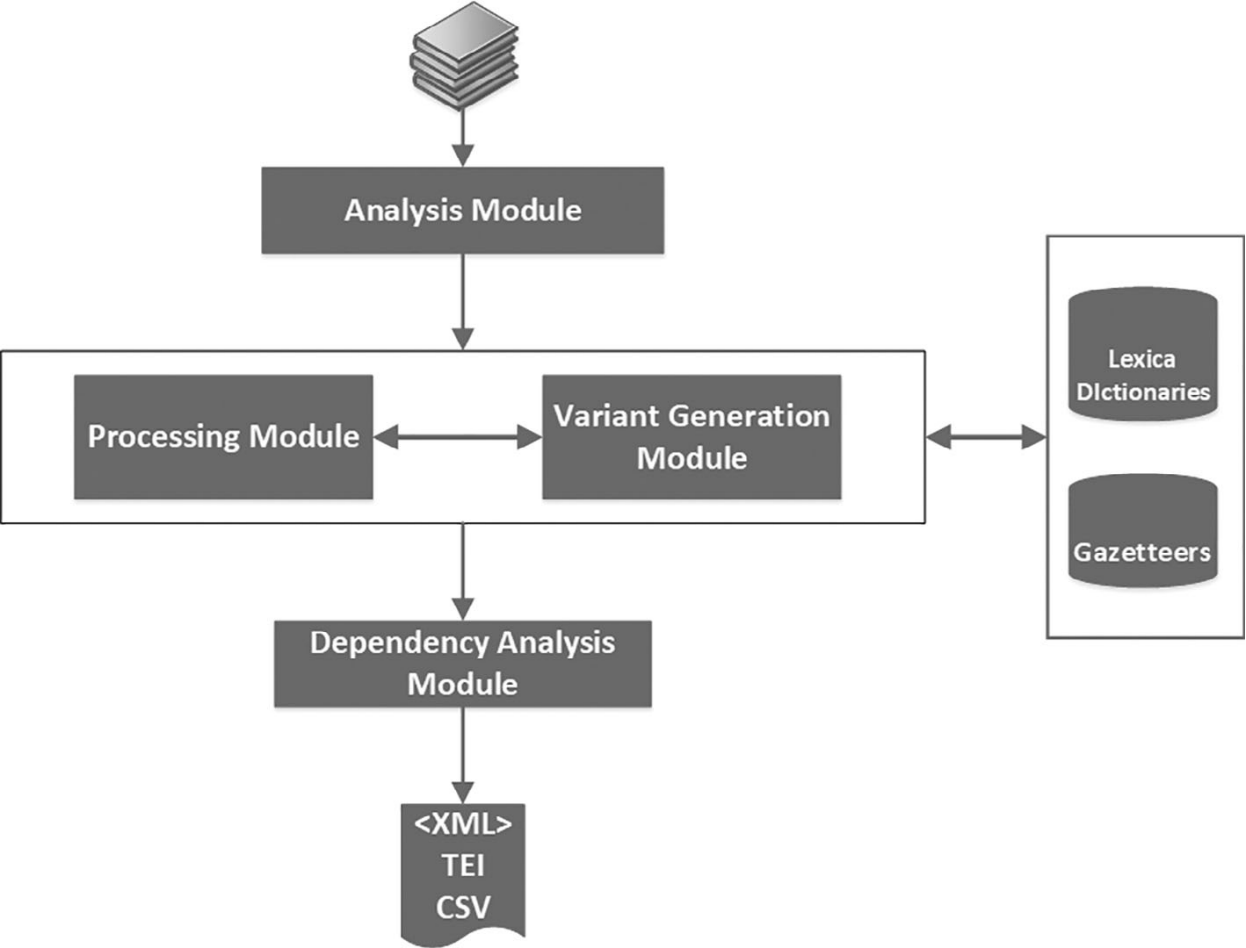
System architecture

The system was conceived with a modular architecture, depicted in Figure 3.

1. **Analysis Module:** Performs a lexical analysis of the text to identify entity candidates or text‐regions likely to contain named‐entities. Upon recognition of a candidate or relevant text‐span, this is passed on to the Processing Module and the subsequent modules.
2. **Processing Module:** Parses the text‐regions identified in the previous step, annotating in these regions named entities and their types.
3. **Variant Generation Module:** When out‐of‐vocabulary items are found in the previous steps, variants are generated for them to find candidate matches in the system's lexical resources (gazetteers, lexica).
4. **Dependency analyzer:** entity attributes are attached to entities thanks to a dependency parse.

### 3.1 *Analysis module*

The module takes medieval Spanish text as its input and consists in a lexical analyzer comprising a set of regular expressions. These expressions were created based on human analysis of medieval corpora to determine typical entity structures and entity‐contexts. The regular expressions, thus, represent lexical and character‐level patterns typical for named entities in the medieval text, that is, patterns based on lexical cues or orthographic cues like punctuation or character case. The analysis module starts processing the input text from its first character onwards. When a match is found for one of the regular expressions, the matched text‐span is considered as containing an entity candidate, or potentially several candidates, depending on the expression matched. The match is then passed on to the processing module. Based on contextual cues specific to each entity‐type, the processing module will parse this text‐span in a type‐specific manner. The processing module may also additionally parse other tokens in the text beyond the original text‐span fed to it; this will depend on the actions determined by the module to be contextually relevant. Once the processing module has completed the processing triggered by the text‐span passed to it, the sequential treatment of the text returns to the lexical analysis module, at the position where the processing module has left off.

We use a set of only five expressions. The first two (RE1 and RE2) model character‐case information and rules RE3 through RE5 are geared towards entities with a more complex structure.

**RE1. Uppercase‐initial tokens:** Words starting with a capital letter shall be recognized and isolated to be processed as possible entity candidates. Means were implemented for sentence‐initial sequences to be treated differently, since they could naturally have an initial capital irrespective of entity status.

**RE2. Lowercase‐initial tokens:** Tokens starting in lowercase are recognized and passed on to the processing module, which will then use such tokens as either entity candidates or contextual cues for entity detection and classification. Lowercase tokens or token sequences can indeed represent entities in medieval text, given irregular use of capitalization. Ambiguity problems may also arise, e.g., *granada*, which can either be a fruit or a city; such problems will be addressed by the processing module.

**RE3. Enumerations of words starting with a capital letter:** This expression was created since in medieval texts there is a great proliferation of enumerations of names, e.g. place names or person names, without punctuation marks between words. The expression will identify text‐spans likely to contain such enumerations.

**RE4. Prepositional phrase concatenation:** This expression captures a typical context in medieval text: chained prepositional phrases (PP), as in the underlined substring of the sequence *rey de Castilla de Leon de Valencia* (*king of Castille of Leon of Valencia*). These PP usually complement a noun and have no intervening punctuation. In the sequence just mentioned, the PPs are locative phrases containing places defining the extent of the king's realms.

**RE5. Forms of address and other triggers:** This rule detects one or several names that start with triggers specific to an entity‐type. E.g. forms of address like *don*, *donna* (*Mr*., *Mme*), terms indicating an authority role (e.g. *rey* for *King*) or an organization, e.g. *Orden* (*Order*).

### 3.2 *Processing module*

The processing module is designed to carry out the processing of text spans matched by the analysis module (names, noun phrases, and other structures) to identify named entities within them. The module operates as a state machine, where the processing of each type of structure is carried out according to the current and previous state.

#### 3.2.1 *Architecture and resources*

The processing module resolves ambiguities using contextual information, aided by lexical resources.^2^ Depending on different cues from the context under processing, besides the previous state, the module determines its current state and adjusts its processing behavior accordingly. Entity candidates fed to the processing module are checked against the following resources:

1. **Gazetteer** for the identification of place names: This is based on Old Spanish resources in the Freeling NLP suite^3^ besides on the Pleiades^4^ and Geonames gazetteers.^5^ The system's gazetteer gets enriched as new texts, containing previously unencountered names or variants, are processed by the system, and thanks to expert validation of these new names. The Freeling dictionary for Medieval Spanish has a small number of errors resulting from the automatic application of generation rules, affecting less than 1% of the lemmas (Sánchez‐Marco, Boleda, & Padró, 2011, p. 6). However, this error rate did not pose problems in our processing.
2. **Dictionaries** of proper nouns, common nouns, names of saints, and organizations. As mentioned for place names, new items are added and validated as new texts are processed by the system.
3. **Entity‐trigger lexica**: These are domain‐specific lexica that help identify entities of a given type. Since the processing module operates as a state machine, these lexica also help determine the current state. The lexica include, among other triggers, nobility titles and forms of address for the person type, and locative phrases for the geographical location type.
4. **Verb subcategorization** dictionary: Contains verb entries, along with stem and subcategorization information, that is, which arguments and (prepositional) complements the verbs take. These verbs are used to identify locative contexts and for dependency parsing. The dictionary was custom‐built for this application, in the absence of a similar resource for Old Spanish.^6^

If a match for an entity candidate is found among the lexical sources, the candidate is tagged for the entity type. When no matches are found, the candidate is passed on to the variant generation module to see if morphophonological variants based on the candidate do have a match in the lexical resources.

Once entities have been recognized via matching candidates (or their variants) against lexical resources the text span for the entity, its type, position, and length, besides metadata like the data source the entity was found in, or geolocation information.

#### 3.2.2 *Entity‐type‐specific contexts*

Named entities in medieval Spanish tend to follow a number of morphosyntactic patterns, and the same patterns can be found in different entity types. The role of character‐case cues in detecting entities is limited, given unstable orthography. However, several contextual cues help in delimiting and typing entities. Taking this into account, to detect entities and their types, several generic polymorphic text‐processing functions have been defined. The implementation of each function is specific to each entity type. Put differently, the implementation applying for each candidate depends on the state in which the state machine finds itself when processing the candidate. The state is determined based on some cues in the candidate and its context, besides the preceding state. Candidate disambiguation depends thus on the system state for each candidate. The cues used to determine the state include prepositions, adverbs, morphosyntactic patterns, and subcategorization information about the verbal system in Medieval Spanish. The possible states and the cues that allow entering and exiting each are described in the following contexts. The contexts described make the system enter a state named after the context (e.g., the locative context triggers a locative state and so on).

1. *General* context
  The general context corresponds to the initial state when processing starts. It is also the context or state to which processing returns upon reaching a sentence boundary.
2. *Locative* context
  This context is identified by the analysis of morphosyntactic patterns based on resources that capture verb subcategorization in Medieval Spanish. These resources were created for our system, according to the descriptive Old Spanish's grammar (García‐Miguel Gallego, 2006) and locative Spanish's complements (Barrajón López, 2015; Jlassi, 2015). These resources are used to determine whether a prepositional complement can be taken to represent a location or not. Certain adverbs are also used to identify this context.
3. *Saint* context
  This context is determined by the identification of adjectives such as *sancto*, *santus*, *santo*, or *santo*, followed by proper nouns. It is common to find place names with names of saints, and religious buildings featuring the name of a saint. If the previous state was locative, the candidate will be disambiguated as part of a place name, rather than as the name of a saint.
4. *Authority* context
  This context is determined through the identification of nouns used for introducing authorities such as *king or commander* (*rey*, *comendador*). These nouns may be followed by phrases complementing them, or directly by the name of the authority. Prepositional phrases like (underlined) *rey de Castilla (King of Castile*) are resolved as a reference to a place associated with this authority. Additional noun phrases can be understood as nicknames, for example, the underlined sequence in *Juana la Loca* (*Joanna the Mad*). Note that authority names can also be part of a place name. However, the state machine will remain in a locative context, which will allow disambiguating the authority reference as part of the place name.
5. *Form of address* context
  The triggers that define this context are common forms of address like *don*, *donna*, *senyor* (*Mr*., *Mrs*., *Mr*.). The resulting entity type is person name. Phrases complementing the name are treated along similar lines as in the authority state (above).
6. *Building* context
  Many of the names of places identified in the texts refer to buildings or landmarks. The identification of nouns such as *iglesia*, *monasterio* (*church*, *monastery*), and others make it convenient to transition to a state in which processing is fast given its context's simplicity. Besides, in a building context, accepting as an entity a candidate that has not been found in the system's lexical resources is unlikely to result in an incorrect tagging.
7. *Geography* context
  In this context, the recognition of place names related to geographical features is sought. The processing state is entered after the identification of nouns representing such features, like *río* or *monte* (*river*, *mountain*). The change to this state simplifies the processing of this type of geographical entities and facilitates treating previously unseen candidates.
8. *Organization* context
  This context is entered based on sequences that refer to organizations, such as the underlined part in *orden de Calatrava* (*Order of Calatrava*).

#### 3.2.3 *Processing functions*

As stated above, several generic processing functions were implemented to delimit and type entity candidates. They are polymorphic functions with a specific implementation for each state. The architecture thus created is easily extensible to deal with new entity types. The processing functions are as follows.

F1. **checkLowerCaseWord**. It processes lowercase words. For the task at hand, most lowercase terms can be ignored, except for two types of items: First, words that are relevant as contextual cues, and for which we created lexica. Second, lowercase words that may be proper nouns written in a non‐standard manner given the orthographic instability in medieval Spanish. Lexica are also available for these.
F2. **checkCapitalizeWord**. It processes uppercase words recognized by the lexical analyzer. Words with an initial capital are checked against the various dictionaries and the gazetteer, and they are tagged in a suitable way according to the context. Sentence‐initial words are treated differently to avoid erroneously tagging them as entity candidates.
F3. **wordListProcessing**. It processes word lists that mostly contain capitalized words, although they can also contain connectors. It is typical for these sequences not to have punctuation that may help delimit entities within the sequence; this is a typical feature in Medieval Spanish. The challenge here is to correctly segment entities within these spans of “undelimited” text. To this end, connectors are taken into account when available. In all cases, n‐grams up to size three are generated for tokens in the sequence. These n‐grams are checked against the system's data sources to validate them as possible entity candidates.
F4. **withPrepositionComplementProcessing**. This function processes sequences of concatenated prepositional phrases, without intervening punctuation. The challenge is to identify which tokens attach syntactically to each preposition.
  N‐grams for the sequence are computed and checked against the lexical resources, as an attempt to identify multi‐token entity candidates.
F5. **NounPhraseWithPrepositionComplementProcessing**. This function processes noun phrases and their attached prepositional complement in cases where there is no attachment ambiguity caused by concatenated prepositional phrases.

If the processing module is unable to parse a text spans using its lexical resources, it interacts with the variant generation module. This action creates variants for the relevant tokens to see if they can then be found in the lexical resources. If even after variant generation no matches are found in the system's lexical resources, the processing module will output entity candidates with a flag that can be exploited for manual revision of the output by experts.

### 3.3 *Variant generation module*

Medieval texts show considerable orthographic instability, given both lacks of standardized orthography and diachronic evolution in Spanish throughout the period. For these reasons, it is common for textual variants to not be present in a system's lexical resources, which makes it more difficult to process them. To address this challenge, variant generation procedures were implemented. The goal of these procedures is to determine if textual sequences not found in the system's lexical resources can be considered as variants of terms available in the resources (lexica and gazetteers). We describe here the variant generation and candidate selection processes.

Two sets of rules were created, to model the diachronic evolution of Spanish in phonetic and morphological terms, based on (Lapesa, 2005). One set captures historical variants' evolution towards modern forms. The other set goes in the opposite direction: it reconstructs possible historical variants starting from modern forms. Having rules that cover both directions improved results over covering one direction only.

Variants are generated for single‐token and multi‐token sequences. All tokens and token sequences that the processing module was not able to classify as a named entity are subjected to variant generation. The generated variants are looked up against the system's lexical resources, taking into account single‐token and multi‐token terms in the resources. Variant generation, which helps deal with out‐of‐vocabulary items in the tool, is performed before dependency parsing, and it can also help lessen the impact of scribal errors in processing.

Rules are applied in an ordered cumulative manner, that is, the output of each rule feeds the next rule, helping detect variants that have undergone several transformations in their historical evolution. About 80 rules were created. Around half of them encode simple transformations that take place regardless of context, whereas the other half take into account contextual information. Several examples of the rules created are given in (Lapesa, 2005). Contexts (using Java regular expression syntax) and replacement expressions for variant generation are given in the GitHub repository.^7^

**FIGURE 4:**
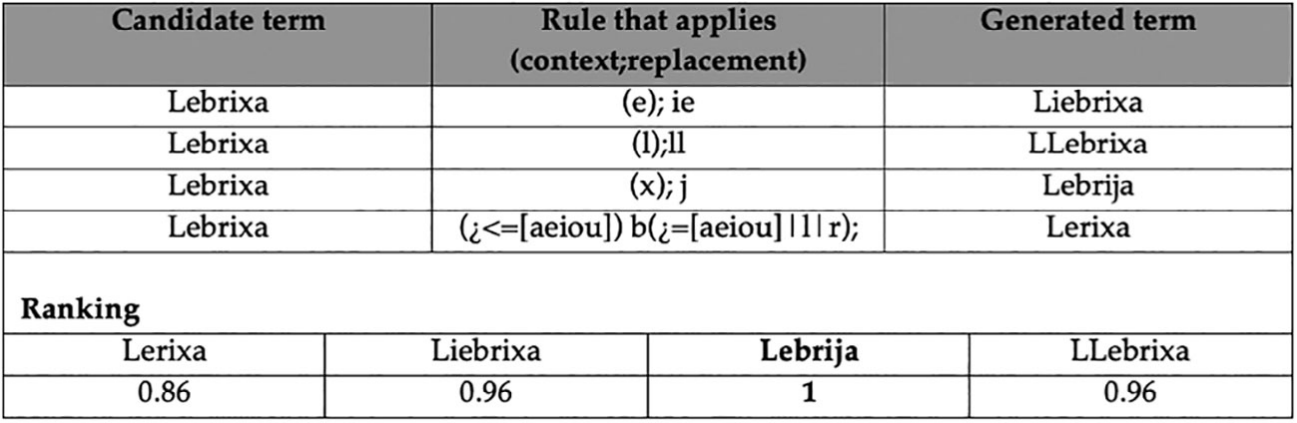
Example of variant generation and ranking (selected variant is bolded)

The variants generated were ranked based on a measure of distance to terms in the lexical resources. The ranking function relies on the Levenshtein edit distance (Damerau, 1964), as well as on the Dice coefficient (Dice, 1945) computed over character bigrams. As per the results of the ranking function, the term in the lexical resources that is closest to one of the variants is selected as the intended term for the variant. A variant can be at equal distance to a term of two different entity‐types in the lexical resources. The state of the processing module will determine which entity type to select. Previously unattested variants for which a term was chosen from the lexical resources are added to the resources as a possible realization of that term, to avoid having to generate the variants in the future (Figure 4).

### 3.4 *Dependency analysis module*

Medieval texts tend to provide information about person names or authorities, like family origin or jurisdiction. Moreover, entities often occur within complex proper noun enumerations. All this information can be relevant for humanities scholars. To provide this information automatically to our users, we implemented a dependency analysis that identifies these structural relations. We describe here the types of dependencies covered, as well as the parsing method, along with some example results showing the challenges involved and our solution for them.

Our dependency parsing identifies entity attributes or relations that may exist between entities embedded in a larger entity. The dependency typology is based on Álvarez et al. (2020). Family relations are identified, as well as honorifics or authority titles related to a person name. The regions over which a ruler's authority extends are also attached to that person via dependencies. As defined in Álvarez et al., we take advantage of TEI syntax to serialize the attributes and relations detected by our dependency parsing. TEI was chosen as this format has wide acceptance in the Humanities community. Besides, XML‐TEI syntax can naturally encode a dependency graph.

Our parser implements Covington's *List‐based search with uniqueness* algorithm (Covington, 2001). It parses by constructing two lists, the list of words that have not yet been analyzed, and the list of words that lack heads. It first searches for dependents, trying to attach them to their heads in an eager manner iterating over the text from left to right. The parser allows a recursive analysis of embedded roles and other embedded entities.

**FIGURE 5:**
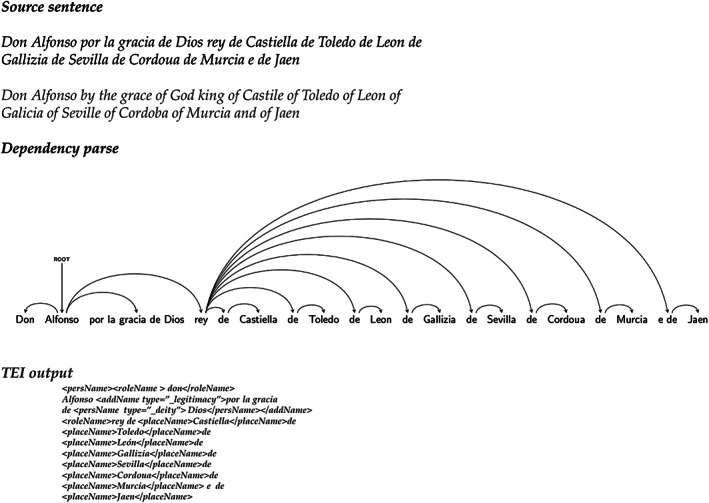
For the source sentence on top, our system provides the dependency parse in the middle. It then serializes the information as text encoding initiative (bottom)

Figure 5 shows a complex text structure example typical for our corpora (top), the dependency parse produced by our system (mid), and our TEI output (bottom).

**FIGURE 6:**
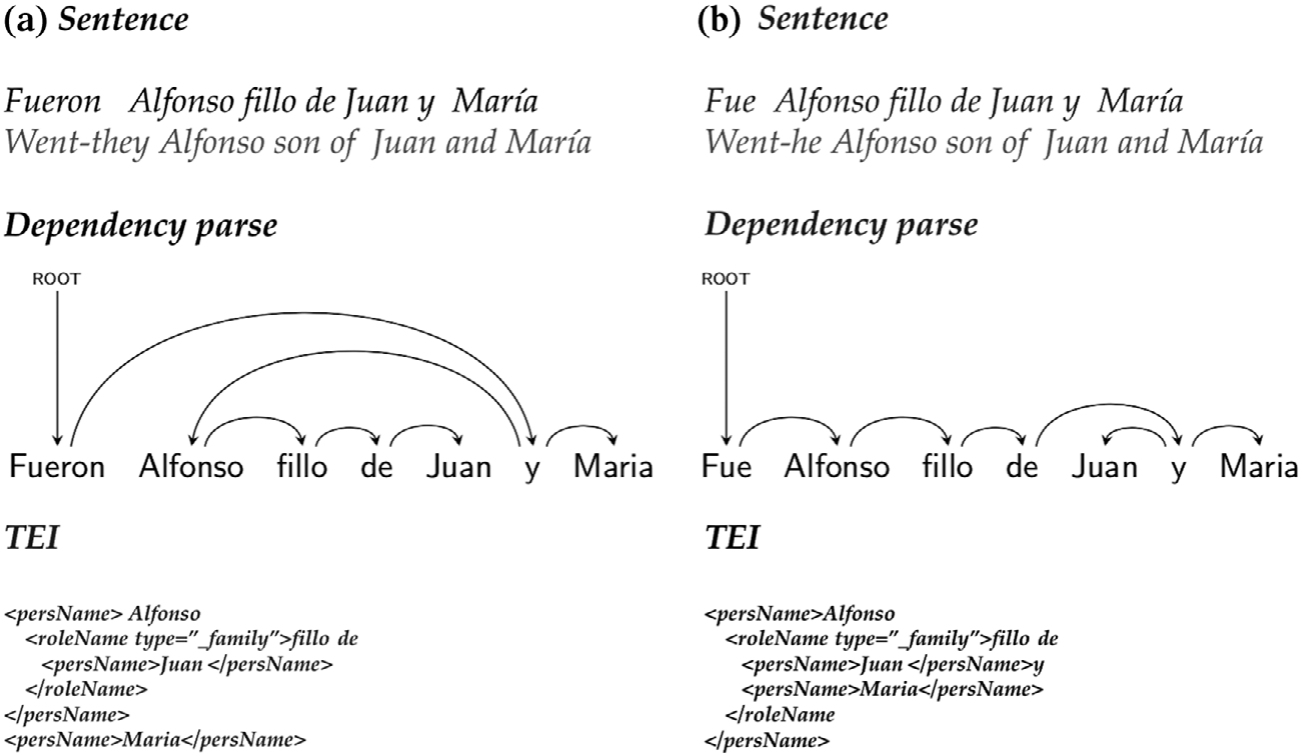
Solving attachment ambiguities in copulative structures. Verb number allows the dependency parser finds the correct solution in each case

Figure 6 illustrates how verb number information can be used to disambiguate attachment in copulative structures. In Figure 6a, a plural verb form leads the parser to attach *Maria* directly to the verb (both *Alfonso* and *Maria* are part of the subject, justifying the plural verb form). In Figure 6b, *Maria* is coordinated with *Juan* and attached to the copulative conjunction, as this is the only choice compatible with a singular verb form.

## 4 EVALUATION AND DISCUSSION

A manually annotated reference corpus was created to be able to evaluate the automatic tagging. This reference corpus consisted of 64,689 words covering different medieval texts and genres (epic poems, legal documents, picaresque novel, dramatic texts) that ranged between the 12th and the 15th century. To ensure the consistency and coherence of the annotation process, a set of annotation guidelines was developed, according to which human annotators carried out their work. The guidelines included a description of the annotation criteria to be followed, illustrated with real examples extracted from the evaluation corpus.^8^

Two annotators carried out the annotation work. The complete reference corpus was manually annotated in XML‐TEI format by a linguist, according to the annotation guidelines. A total of 3,974 named‐entities was tagged. An amount of text covering approximately 50% of those entities (2,054 items) was manually annotated by a second linguist so that we could compute inter‐annotator agreement. This score was measured with the kappa coefficient (Artstein & Poesio, 2008; Carletta, 1996), obtaining a kappa value of 0.802 (*N* = 2054, *K* = 2). We consider that this kappa value suggests that the manual annotations are reliable, based on discussions on kappa values (Krippendorff, 1980; Landis & Koch, 1977).

Two evaluation tasks were defined. First, a standard NER and Classification (NERC) or sequence labelling task, where the goal is to correctly delimit NEs and assign them the right type, the second task can be seen as an entity‐attribute detection task. We describe both in the following section.

### 4.1 *Named‐entity task*

The system's output was evaluated against the manual reference, overall, and per entity type. A positive result was defined as an exact match in terms of annotated span (start and end offset), type for the span across reference and system results. The evaluation metrics were precision, recall and F1, with the usual definitions, as follows:

1. Precision: number of entities correctly tagged divided by the number of entities output by the automatic tagging system.
2. Recall: number of entities correctly tagged divided by of the number of entities in the manually annotated reference results.
3. F1: The harmonic mean of precision and recall, which ranges between 0 and 1, with 1 being the best possible value.

Overall and per‐type results are shown in Table 1. Since medieval Spanish kept evolving over several centuries without a fixed orthographic standard, and we wanted to assess our system's performance with the different historical varieties, the corpus was divided into 50‐year periods, and the overall F1 was computed per period (see Table 2).

**TABLE 1:** Medieval Spanish Named Entity Recognition Classification. Precision, Recall, F1, overall and per entity‐type, for the complete reference corpus

Entity type	Precision	Recall	F1
Overall	0.83	0.72	0.77
*placeName*	0.78	0.73	0.75
*persName*	0.84	0.79	0.81
*orgName*	0.93	0.81	0.87
*roleName*	0.87	0.65	0.74

**TABLE 2:** Medieval Spanish Named Entity Recognition Classification. F1 per period in the reference corpus

Period (years)	F1
1,150–1,200	0.85
1,200–1,250	0.73
1,250–1,300	0.89
1,300–1,350	0.76
1,350–1,370	0.63
1,400–1,500	0.67

As Table 1 shows, F1 values range from 0.74 to 0.87 depending on the entity type. The F1 overall score is 0.77. We consider these results satisfactory because of the variability in spelling and usage shown in medieval Spanish, which makes it challenging to design automatic linguistic analysis tools for this variety. To calculate the metrics of different entities' types and the overall scores, a micro‐average was used, since the number of entities was not distributed uniformly in the corpus (Cornolti, Ferragina, & Ciaramita, 2013; Tjong Kim Sang, 2002; Tjong Kim Sang & De Meulder, 2003).

The higher F1 score for orgName is explained by the fact that in medieval texts variety for this entity type is limited and our lexica already contained many of the organizations found in the corpus so that their identification was less challenging than for other types. Also note that following (Sekine & Nobata, 2004), our system annotates the names of historical peoples (the Trojans, the Goths, so on.) as organization names. Such cases are part of our lexical resources and their detection is easy.

Better results for the most archaic varieties reflect the fact that custom rule‐sets were created to handle archaic forms, based on morphophonological evolution patterns, as described in Spanish historical linguistics works. As regards the periods for which results are lower, note that they were primarily tested on notarial texts. These texts are more difficult, given a large amount of person names and role names which are challenging to segment and impact negatively in the results. Since the application's lexical resources were enriched after testing, based on variants found in the test corpora, it can be expected that results will improve if we test on similar but previously unseen texts of comparable difficulty.

### 4.2 *Attribute detection task*

The goal of this task is to assess to what an extent our procedures to annotate entity attributes were effective. Entity attributes were only evaluated for categories *persName* and *roleName* in our taxonomy. Note that only some of the *persName* and *roleName* instances have attributes in the corpus. We speak of entity “attributes,” however, some of these types of information can be seen as nested entities, as in the case of place names that represent a ruler's jurisdiction (see Table 3).

**TABLE 3:** Types of entity attributes or dependent entities annotated by our system

Attribute type	Text encoding initiative (TEI) label	Attribute meaning	Example (head bolded, attribute underlined)[gloss]
*persName* attributes			
*persRoleHon*	<*roleName* type = “honorific”	Honorifics, forms of address	Don **Alfonsso** [Don **Alfonsso**]
*persRoleAuth*	<*roleName* type = “_authority”	Authority or professional roles	Rey **Alfonso** [king **Alfonso**] **Alfonsso** Rey [**Alfonso** king]
*persRoleFami*	<*roleName* type = “_family”	Family attributes, when the person name is the head	**Alfonsso** fiio de Johan [**Alfonsso** son of Johan]
*persAddNick*	<*addName* type = “nickname”	Nicknames for a person	**Rodrigo Diaz** el Cid [**Rodrigo Diaz** el Cid]
*persPlace*	<*placeName*>	Place names representing a person's origin	**Diaz** de Biuar [**Diaz** de Biuar]
*roleName* attributes			
*roleAuthPlace*	<*placeName*>	Place representing the jurisdiction for a political role	**Rey** de Castiella **[king** of castile]
*roleAddLegi*	<*addName* type = “_legitimacy”	Legitimacy formulas	**Rey** por la gracia de Dios **[king** by the grace of god]
*roleFamiPers*	<*persName*>	Family attributes, when the family term is the head	Alfonsso **fiio** de Johan [Alfonsso **son** of Johan]

These attributes or nested entities were analyzed via dependency parses in our system. For evaluation, we used an F1 score computed over head‐dependent pairs.^9^ For a positive result, the labels for the head and the dependent had to match the reference. This is equivalent to the dependency type for a given arc matching the reference since dependency types (Table 3) in our typology are defined based on the entity types of the head and dependent. The F1 metric was defined as follows:

1. Precision: number of head‐dependent pairs correctly labeled divided by the number of head‐dependent pairs output by the system.
2. Recall: number of head‐dependent pairs correctly labeled divided by the number of head‐dependent pairs in the reference.
3. F1: The harmonic mean of precision and recall, which ranges between 0 and 1, with 1 being the best possible value.

The dependencies or attributes we evaluated are shown in Table 3. As mentioned, only some entities (529) had attributes. Among these entities, 361 were *persName* and 168 were *roleName*. The different metrics and overall scores (row *all*) are micro‐averaged, as we did in NERC evaluation (see Table 1).

For computing the F1 scores (Table 4), we grouped items according to whether the attribute/dependent entity is governed by a *persName* or by a *roleName*. The reason for doing so is that the number of items per category for some of the types examined would be too small for its separate evaluation to be interesting. Our groupings are justified since we want to evaluate the extent to which our system can identify the information provided in medieval texts about *persName* and *roleName* entities. These texts sometimes add rich information about such entity types, like family or geographical origin, professional or administrative roles, rulers' jurisdiction, so on.

**TABLE 4:** Attribute extraction for persName and roleName heads. Precision, Recall, F1 assessed with an exact match of head and dependent attribute

Head	N	Precision	Recall	F1
*persName*	361	0.96	0.84	0.89
*roleName*	168	0.80	0.65	0.71
All	529	0.82	0.70	0.75

## 5 CONCLUSIONS

A system to detect named entities in different varieties of medieval Spanish was presented, which annotates against a custom named entity taxonomy geared towards medieval texts, which we also created for our project. Orthographic and lexical variability in medieval Spanish were addressed thanks to variant generation and normalization; the system can handle unnormalized medieval text. The system uses dependency parsing to annotate person name attributes like origin or family information, besides role attributes (e.g., treatments, professional, or political functions). As such, the system is providing information about medieval entities that was not available with prior NER tools for medieval Spanish. A quantitative evaluation of both NER and attribute detection was satisfactory. Our tool is a symbolic system, with annotation rules that could be modified by domain experts, that is, humanists studying medieval texts. The system outputs TEI annotations, as this is a widely used format in the humanities, besides logs that would allow users to assess result quality and trace error sources. To make the tool yet more usable by humanities scholars, a user interface exploiting the tool's results and giving access to its resources for easy modification by domain experts is planned.

## 6 SOFTWARE

All the source code and corpus are available at GitHub repository: https://github.com/linhd-postdata/HisMetag. We have included the docker version of the tool and the tool's API.

## Endnotes

^1^
http://nlp.lsi.upc.edu/freeling/.

^2^ The resources are at the following location in the tool's GitHub repository: https://github.com/linhd-postdata/HisMetag/tree/master/hismetag/src/main/resources/dataFiles.

^3^
https://github.com/TALP-UPC/FreeLing/tree/master/data/es/es-old.

^4^
https://pleiades.stoa.org/downloads.

^5^
http://download.geonames.org/export/dump/.

^6^ The dictionary can be found in the tool's GitHub repository, at https://github.com/linhd-postdata/HisMetag/tree/master/hismetag/src/main/resources/dataFiles/verbos.txt. It lists the prepositions introducing complements governed by certain verbs (in the third column), besides other information regarding the contexts in which the verb is likely to appear (e.g., a locative context for motion verbs is tagged as “PL” in the last column).

^7^ For changes intended to map older variants to newer variants of a word, the rules are at https://github.com/linhd-postdata/HisMetag/blob/master/hismetag/src/main/resources/dataFiles/rules.txt For changes intended to map later variants to their earlier forms, the rules are at https://github.com/linhd-postdata/HisMetag/blob/master/hismetag/src/main/resources/dataFiles/reverserules.txt. In all cases, the separator between the context and replacement is a semi‐colon.

^8^ The guidelines now exist as an internal report, but can be made publicly available.

^9^ As only a subset of tokens in the text show entity‐relevant dependencies, we decided against evaluating with a more typical measure for dependency parsing such as Labeled Attachment Score (or LAS) (Bucholz & Marsi, 2006), which is used when all tokens in the text take part in a dependency that it is relevant to evaluate.

## References

[asi24399-bib-0001] Agerri, R. , & Rigau, G. (2016). Robust multilingual named entity recognition with shallow semi‐supervised features. Artificial Intelligence, 238, 63–82. 10.1016/j.artint.2016.05.003

[asi24399-bib-0002] Albaigès, J. M. , & i Olivart, J. M. A. (1995). Enciclopedia de los nombres propios. Madrid, Spain: Planeta Publishing Corporation.

[asi24399-bib-0048] Álvarez Mellado, E. , Díez‐Plata, M. , Ruíz Fabo, P. , Bermúdez, H. , Ros, S. , & González‐Blanco, E. (2020). TEI‐friendly annotation scheme for medieval named entities. A case on a Spanish medieval corpus. Language Resources and Evaluation. 10.1007/s10579-020-09516-2 PMC8550670 34776810

[asi24399-bib-0003] Alex, B. , Byrne, K. , Grover, C. , & Tobin, R. (2015). Adapting the Edinburgh Geoparser for historical Georeferencing. International Journal of Humanities and Arts Computing, 9(1), 15–35. 10.3366/ijhac.2015.0136

[asi24399-bib-0004] Alvar, M. (1996). Manual de dialectología hispánica: el español de España (Vol. 1). Madrid, Spain: Editorial Planeta.

[asi24399-bib-0005] Artstein, R. , & Poesio, M. (2008). Inter‐coder agreement for computational linguistics. Computational Linguistics, 34(4), 555–596.

[asi24399-bib-0006] Barrajón López, E. B. (2015). La función semántica del complemento preposicional nocional y local. Revista de Investigación Lingüística, 17, 11–30.

[asi24399-bib-0007] Borin, L. , Kokkinakis, D. , & Olsson, L.‐J. (2007). Naming the past: Named entity and animacy recognition in 19th century Swedish literature. Proceedings of the Workshop on Language Technology for Cultural Heritage Data (LaT‐ECH 2007), 1–8. http://hnk.ffzg.hr/bibl/ACL2007/LaTeCH/LaTeCH-2007.pdf#page=11

[asi24399-bib-0047] Buchholz, S. , & Marsi, E. (2006). CoNLL‐X shared task on multilingual dependency parsing Proceedings of the Tenth Conference on Computational Natural Language Learning, New York: Association for Computational Linguistics; 149–164.

[asi24399-bib-0008] Cano‐Aguilar, R. (2004). Historia de la lengua española. Ariel. https://dialnet.unirioja.es/servlet/libro?codigo=10913

[asi24399-bib-0009] Carletta, J. (1996). Assessing agreement on classification tasks: The kappa statistic. Computational Linguistics, 22(2), 249–254.

[asi24399-bib-0010] Chinchor, N. , & Marsh, E. (1998). Muc‐7 information extraction task definition. Proceeding of the seventh message understanding conference (MUC‐7), appendices, 359–367. http://www.aclweb.org/anthology/M/M98/M98-1027.pdf

[asi24399-bib-0011] Collier, N. , Ruch, P. , & Nazarenko, A. (Eds.). (2004). JNLPBA'04: Proceedings of the international joint workshop on natural language processing in biomedicine and its applications. Stroudsburg, PA: Association for Computational Linguistics.

[asi24399-bib-0012] Cornolti, M. , Ferragina, P. , & Ciaramita, M. (2013). A framework for benchmarking entity‐annotation systems. Proceedings of the 22nd International Conference on World Wide Web, 249–260. http://dl.acm.org/citation.cfm?id=2488411

[asi24399-bib-0013] Cortes, C. , & Vapnik, V. (1995). Support‐vector networks. Machine Learning, 20(3), 273–297. 10.1007/BF00994018

[asi24399-bib-0014] Covington, M. A. (2001). A fundamental algorithm for dependency parsing. *In* Proceedings of the 39th Annual ACM Southeast Conference, 95–102.

[asi24399-bib-0015] Cucchiarelli, A. , & Velardi, P. (2001). Unsupervised named entity recognition using syntactic and semantic contextual evidence. Computational Linguistics, 27(1), 123–131.

[asi24399-bib-0016] Damerau, F. J. (1964). A technique for computer detection and correction of spelling errors. Communications of the ACM, 7(3), 171–176.

[asi24399-bib-0017] Desmet, B. , & Hoste, V. (2014). Fine‐grained Dutch named entity recognition. Language Resources and Evaluation, 48(2), 307–343.

[asi24399-bib-0018] Dice, L. R. (1945). Measures of the amount of ecologic association between species. Ecology, 26(3), 297–302. 10.2307/1932409

[asi24399-bib-0019] Ehrmann, M. , Colavizza, G. , Rochat, Y. , & Kaplan, F. (2016). Diachronic Evaluation of NER Systems on Old Newspapers. Proceedings of the 13th Conference on Natural Language Processing (KONVENS 2016)), 97–107. https://infoscience.epfl.ch/record/221391

[asi24399-bib-0020] García‐Miguel Gallego, J. M. (2006). Los complementos locativos. *Sintaxis histórica de la lengua española*, *Vol* *1*, *Tomo 2*, 2006 *(Primera parte*, *La frase verbal)*, *ISBN 968–16–7739‐0*, *págs* *1253–1338*, 1253–1338. https://dialnet.unirioja.es/servlet/articulo?codigo=2002540

[asi24399-bib-0021] Grishman, R. (2015). Information extraction. *Intelligent Systems* . IEEE, 30(5), 8–15.

[asi24399-bib-0022] Grishman, R. , & Sundheim, B. (1996). Message understanding Conference‐6: A brief history. COLING, 96, 466–471 http://www.alta.asn.au/events/altss_w2003_proc/altss/courses/molla/C96-1079.pdf

[asi24399-bib-0023] Grover, C. , Givon, S. , Tobin, R. , & Ball, J. (2008). Named Entity Recognition for Digitised Historical Texts. *Proceedings of the Sixth International Conference on Language Resources and Evaluation (LREC'08*). LREC, Marrakech, Morocco. http://www.lrec-conf.org/proceedings/lrec2008/pdf/342_paper.pdf

[asi24399-bib-0024] Grover, C. , Tobin, R. , Byrne, K. , Woollard, M. , Reid, J. , Dunn, S. , & Ball, J. (2010). Use of the Edinburgh geoparser for georeferencing digitized historical collections. Philosophical Transactions of the Royal Society of London A: Mathematical, Physical and Engineering Sciences, 368(1925), 3875–3889. 10.1098/rsta.2010.0149 20643682

[asi24399-bib-0025] Hobbs, J. R. , Appelt, D. , Bear, J. , Israel, D. , Kameyama, M. , & Tyson, M. (1993). Fastus: A system for extracting information from text. Proceedings of the Workshop on Human Language Technology, 133–137. http://dl.acm.org/citation.cfm?id=1075701

[asi24399-bib-0026] Huang, Z. , Xu, W. , & Yu, K. (2015). Bidirectional LSTM‐CRF models for sequence tagging. ArXiv: abs/1508.01991.

[asi24399-bib-0027] Iglesias Moreno, M. E. , Aguilar‐Amat, P. A. , & Sánchez Cuadrado, S. (2014). Primera aproximación para la extracción automática de Entidades Nombradas en corpus de documentos medievales castellanos. Janus: Estudios Sobre El Siglo de Oro, 1, 229–238.

[asi24399-bib-0028] Jlassi, E. (2015). Semántica de las preposiciones en la función de aditamento en los textos de Alfonso X. *Estudio comparativo* http://digibuo.uniovi.es/dspace/handle/10651/32111

[asi24399-bib-0029] Krippendorff, K. (1980). Content analysis: An introduction to its methodology, California, USA: Sage Publications.

[asi24399-bib-0030] Lafferty, J. D. , McCallum, A. , & Pereira, F. C. N. (2001). Conditional random fields: Probabilistic models for segmenting and Labeling sequence data In Proceedings of the eighteenth international conference on machine learning (pp. 282–289). New York: ACM http://portal.acm.org/citation.cfm?id=655813.

[asi24399-bib-0031] Landis, J. R. , & Koch, G. G. (1977). The measurement of observer agreement for categorical data. Biometrics, 33, 159–174. 843571

[asi24399-bib-0032] Lapesa, R. (2005). HISTORIA DE LA LENGUA ESPAÑOLA. Gredos.

[asi24399-bib-0033] Linguistic Data Consortium . (2008). ACE English Entity Guidelines. https://www.ldc.upenn.edu/sites/www.ldc.upenn.edu/files/english-entities-guidelines-v6.6.pdf

[asi24399-bib-0034] Nadeau, D. , & Sekine, S. (2007). A survey of named entity recognition and classification. Lingvisticae Investigationes, 30(1), 3–26.

[asi24399-bib-0035] Padró, L. , & Stanilovsky, E. (2012, May). FreeLing 3.0: Towards Wider Multilinguality. *Proceedings of the Language Resources and Evaluation Conference (LREC 2012)*.

[asi24399-bib-0036] Ringland, N. (2015). Structured Named Entities. https://ses.library.usyd.edu.au/handle/2123/14558

[asi24399-bib-0037] Ritter, A. , Clark, S. , Mausam , & Etzioni, O. (2011). Named Entity Recognition in Tweets: An Experimental Study. *Proceedings of the Conference on Empirical Methods in Natural Language Processing*, 1524–1534. http://dl.acm.org/citation.cfm?id=2145432.2145595

[asi24399-bib-0038] Rosset, S. , Grouin, C. , Fort, K. , Galibert, O. , Kahn, J. , & Zweigenbaum, P. (2012). Structured named entities in two distinct press corpora: Contemporary broadcast news and old newspapers. Proceedings of the Sixth Linguistic Annotation Workshop, 40–48.

[asi24399-bib-0039] Sánchez‐Marco, C. , Boleda, G. , & Padró, L. (2011). Extending the tool, or how to annotate historical language varieties. Proceedings of the 5th ACL‐HLT Workshop on Language Technology for Cultural Heritage, Social Sciences, and Humanities, 1–9. https://www.aclweb.org/anthology/W11-1501

[asi24399-bib-0040] Sekine, S. , & Nobata, C. (2004). Definition, dictionaries and tagger for extended named entity hierarchy Proceedings of the Fourth International Conference on Language Resources and Evaluation, 1977–1980). Lisbon, Portugal: European Language Resources Association (ELRA) http://lrec.elra.info/proceedings/lrec2004/pdf/65.pdf.

[asi24399-bib-0041] Sporleder, C. (2010). Natural language processing for cultural heritage domains. Language and Linguistics Compass, 4(9), 750–768.

[asi24399-bib-0042] Strauss, B. , Toma, B. E. , Ritter, A. , de Marneffe, M. C. , & Xu, W. (2016). Results of the wnut16 named entity recognition shared task.

[asi24399-bib-0043] Thomas, A. , & Sangeetha, S. (2019). An innovative hybrid approach for extracting named entities from unstructured text data. Computational Intelligence, 35(4), 799–826. 10.1111/coin.12214

[asi24399-bib-0044] Tjong Kim Sang, E. F . (2002). Introduction to the CoNLL‐2002 shared task: Language‐independent named entity recognition. *COLING‐02*: *The 6th Conference on Natural Language Learning* 2002 *(CoNLL‐*2002*)* https://www.aclweb.org/anthology/W02-2024

[asi24399-bib-0045] Tjong Kim Sang, E. F. , & De Meulder, F. (2003). Introduction to the CoNLL‐2003 shared task: Language‐independent named entity recognition. Proceedings of the Seventh Conference on Natural Language Learning at HLT‐NAACL 2003, 4, 142–147. http://dl.acm.org/citation.cfm?id=1119195

[asi24399-bib-0046] Volk, M. , Bubenhofer, N. , Althaus, A. , Bangerter, M. , Furrer, L. , & Ruef, B. (2010). Challenges in building a multilingual alpine heritage corpus In Proceedings of the Seventh International Conference on Language Resources and Evaluation LREC'10. Valleta, Malta: European Language Resources Association (ELRA).

